# Review of current 2SLGBTQIA+ inequities in the Canadian health care system

**DOI:** 10.3389/fpubh.2023.1183284

**Published:** 2023-07-18

**Authors:** Dominique Comeau, Claire Johnson, Nadia Bouhamdani

**Affiliations:** ^1^Vitalité Health Network, Dr. Georges-L.-Dumont University Hospital Center, Research Sector, Moncton, NB, Canada; ^2^School of Public Policy Studies, Université de Moncton, Moncton, NB, Canada; ^3^Medicine and Health Sciences Faculty, Université de Sherbrooke, Sherbrooke, QC, Canada; ^4^Centre de Formation Médicale du Nouveau-Brunswick, Université de Moncton, Moncton, NB, Canada

**Keywords:** 2SLGBTQIA+, health care, Canada, health inequities, determinants of health, gender, sexual minority

## Abstract

Gender identity and sexual orientation are determinants of health that can contribute to health inequities. In the 2SLGBTQIA+ community, belonging to a sexual and/or gender minority group leads to a higher risk of negative health outcomes such as depression, anxiety, and cancer, as well as maladaptive behaviors leading to poorer health outcomes such as substance abuse and risky sexual behavior. Empirical evidence suggests that inequities in terms of accessibility to health care, quality of care, inclusivity, and satisfaction of care, are pervasive and entrenched in the health care system. A better understanding of the current Canadian health care context for individuals of the 2SLGBTQIA+ community is imperative to inform public policy and develop sensitive public health interventions to make meaningful headway in reducing inequity. Our search strategy was Canadian-centric and aimed at highlighting the current state of 2SLGBTQIA+ health inequities in Canada. Discrimination, patient care and access to care, education and training of health care professionals, and crucial changes at the systemic and infrastructure levels have been identified as main themes in the literature. Furthermore, we describe health care-related disparities in the 2SLGBTQIA+ community, and present available resources and guidelines that can guide healthcare providers in narrowing the gap in inequities. Herein, the lack of training for both clinical and non-clinical staff has been identified as the most critical issue influencing health care systems. Researchers, educators, and practitioners should invest in health care professional training and future research should evaluate the effectiveness of interventions on staff attitudinal changes toward the 2SLGBTQIA+ community and the impact on patient outcomes.

## 1. Introduction

The determinants of health encompass personal, social, economic, and environmental factors that determine health, such as income and social status, employment and working conditions, education and literacy, childhood experiences, access to health services, racism, culture, biology and genetic endowment, age, sexual orientation, and gender identity (1, 2). Certain health determinants can contribute to health inequities by inciting discriminatory and/or intolerant behaviors. The acronym 2SLGBTQIA+ (Two-Spirit, Lesbian, Gay, Bisexual, Transgender/Trans, Queer/Questioning, Intersexual, Asexual/Aromantic/Agender and all others) is used to describe sexual and gender minorities (3). Individuals of sexual and/or gender minority are often faced with higher rates of adverse health outcomes when compared to the general population; this includes suicide, anxiety, cancer, obesity, and arthritis (4). They are also more likely to engage in harmful health behaviors, such as substance use and risky sexual conduct (4).

2SLGBTQIA+ health disparities can be explained by minority stress theory (5) which postulates that high levels of chronic stress brought on by enacted and/or anticipated stigmatization and discrimination, as well as internalized cis-hetero-normativity (i.e., the presumption that all individuals are inherently cis-gendered and heterosexual) are detrimental to health (6–9). This chronic stress directly impacts physical and psychosocial health and drives avoidance behaviors that impact health. The health of 2SLGBTQIA+ individuals has often been compromised by stigmatization, discrimination, a lack of visibility, and a lack of cultural competency in the health care system (10–21). In addition, the notion of intersectionality explains how 2SLGBTQIA+ individuals may be confronted with multiple interlinked forms of discrimination like cis-hetero-normativity, agism, ableism and racism, that when combined, increase vulnerability to health inequities and lead to inferior clinical outcomes (22–25). In other words, the health equity gap widens increasingly.

Importantly, these risk factors are modifiable when proper action is taken to address them. This involves adequate education for health care professionals (HCP) in matters of 2SLGBTQIA+ specific health needs, cultural competency, and implicit bias training, as well as resolving systemic discrimination and lack of inclusivity within the health care system. The main goal of this review is to present succinct and up-to-date information on the current state of 2SLGBTQIA+ health inequities in Canada. Specific objectives were to (i) present known factors associated with health disparities in the 2SLGBTQIA+ community and (ii) identify existing resources that can be used by Canadian health care institutions to address inequities and therefore, ameliorate the health outcomes of the 2SLGBTQIA+ community. Discrimination, patient care, and access to care, education and training of HCP, and crucial changes at the systemic and infrastructure levels have been identified as main themes in the literature. They will be discussed throughout this review.

## 2. Discrimination

Access to quality health care is a fundamental right that is not always extended to the 2SLGBTQIA+ community because of individual and systemic discrimination. Discriminatory attitudes from HCP are a major problem in the health care system (26–29). Notably, refusal to administer treatment and medication and delaying treatments for 2SLGBTQIA+ individuals are striking examples of individual discrimination by HCP (15, 17, 30–36). Contrastingly to these reports of misconduct, studies have demonstrated an increase in explicitly positive attitudes toward the 2SLGBTQIA+ community from nursing staff and physicians over time (12–14, 37–39). However, despite this rise in explicit positive attitudes, patients remain victims of stigma, discrimination and are denied their civil and human rights (11, 40). It is thus important to distinguish between implicit and explicit biases; even when HCP are committed to providing equal care, this can be undermined by implicit biases operating unconsciously (41–46). These psychological biases are described as the nonconscious or implicit prejudice and stereotypes health care providers hold without knowing it. Even when HCP are committed to providing equitable care, some evidence supports that implicit biases can impact health care providers’ judgment and behavior when interacting with patients from the 2SLGBTQIA+ community (40).

**Figure 1:**
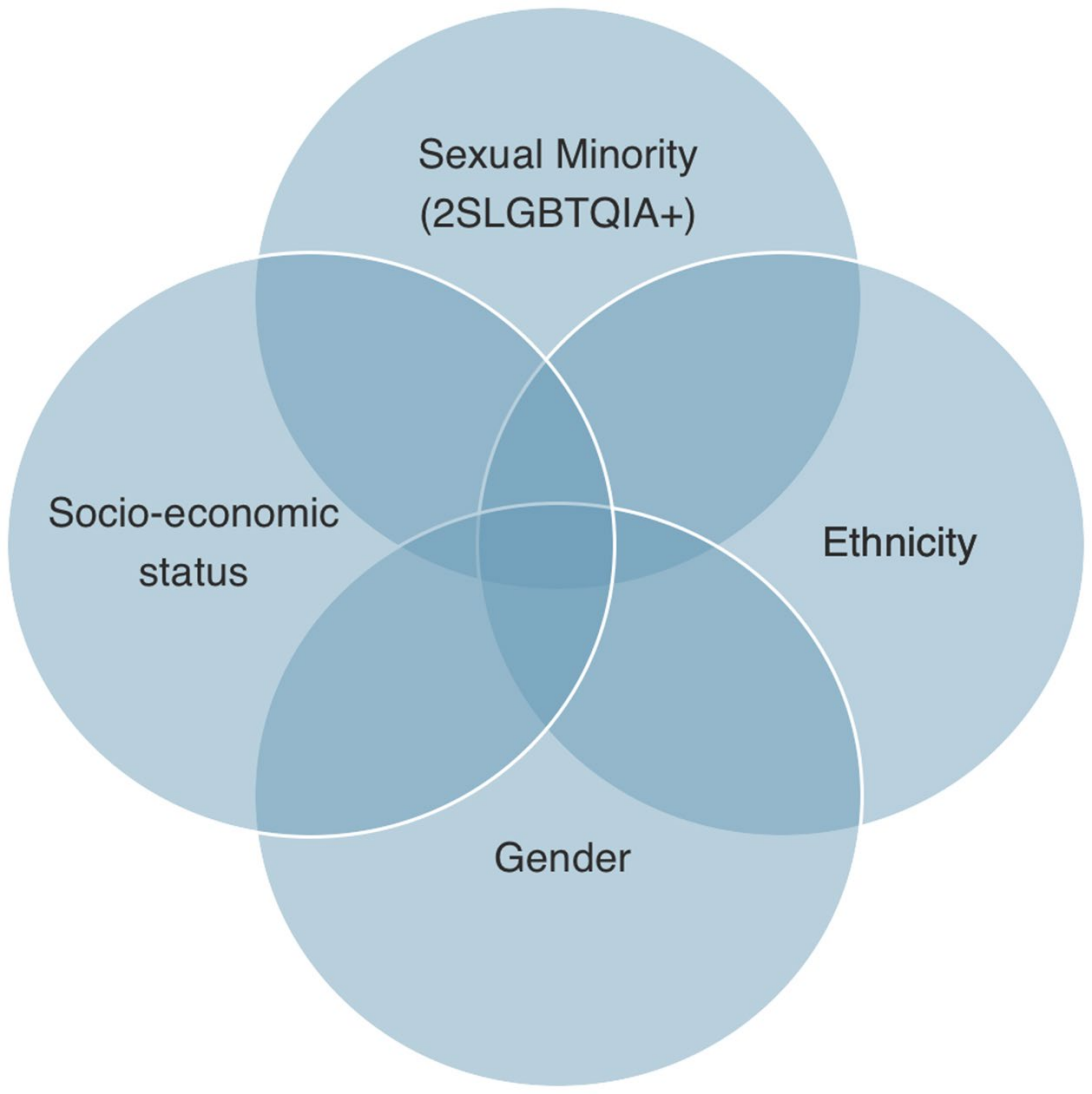
The intersectionality of social determinants of health to illustrate the compounding impact on health disparities.

Discrimination is also found at the systemic level, in forms, processes, language used, medical records, and the physical environment, exacerbating the discriminatory experience within the health care system (4, 47, 48). This leads to delayed treatment, which inevitably has a damaging impact on health (13, 14, 30–33, 36, 49–53). Furthermore, general apprehension and being cognizant of the negative impact that divulgation may have on health leads to non-disclosure of sexual orientation and/or gender identity. Although non-disclosure protects patients from prejudice short term, it has a detrimental impact on health long term, more specifically, on patient care, health, and satisfaction of care (10, 15, 36, 50, 52, 54, 55). Hence, patients are put in a lose-lose situation created by an inequitable health care system that ultimately contributes to health disparities. Stigma and discrimination also lead to unfair access to social and material resources auxiliary to good health like employment, income, housing, education and health care (56). For example, the Trans PULSE Project (a community-based research project that investigated the impact of social exclusion and discrimination on the health of 2SLGBTQIA+ people in Ontario, Canada) found that 40 and 45% of the transgender population surveyed had low-income and un-met health needs, respectively, and bisexual folks experienced food insecurities at double the rate of their heterosexual counterparts (57). These disparities are further exacerbated by race, socio-economic status, gender, and sexual orientation through an intersectional lens (24). These findings can be explained by the intersectionality theory, where the intersection of social health determinants have a compounding impact on health (25). As shown in Figure 1, this theory is often used to correct for health determinants that would otherwise be analyzed separately to assess their direct impact on health instead of looking at how they interact together. In reality, various determinants interact together and compound the impact on an individual’s health. Many studies in this review have illustrated this phenomenon in the 2SLGBTQIA+ community in Canada, where sexual or gender minority status will have an influence on other health determinants directly or indirectly and ultimately impact health.

## 3. Patient care and access to care

The minority stress framework posits that 2SLGBTQIA+ individuals are subject to stressors that have a pernicious effect on physical and psychosocial health (58, 59). Notably, the 2SLGBTQIA+ community disproportionately experiences stigmatization and discrimination which can lead to mental illness (e.g., increased risk of depression, suicidal ideation, anxiety), as well as maladaptive behaviors such as substance abuse and risky sexual behavior that may lead to higher rates of HIV and sexually transmitted infections (54, 60–66). Often, these risk factors are overlooked by clinicians, making it difficult to properly treat patients (67, 68).

In the United States, half of the people diagnosed with HIV are gay and bisexual men (60, 69); these figures are roughly the same in Canada (4). In addition, men who have sex with men are at higher risk of developing anal and genital cancers (70, 71). Lesbian and bisexual women tend to have more binge eating behaviors to deal with social isolation and other pressures related to minority stress (72). This partly explains why lesbian and bisexual women are more likely to be overweight and suffer from obesity, which increases their risk of cardiovascular disease and arthritis compared to the general population (4, 48, 54, 69, 73, 74). Once again illustrating how these increased health risks could be mitigated by reducing the harm associated with minority stress (72). In addition, they also tend to avoid screening for breast cancer, cervical cancer, and sexually transmitted infections due to feelings of distress associated with medical visits (11, 54, 60, 69). Lower screening rates could potentially lead to an increased risk of cancer diagnoses in this population (75, 76) and the development of advanced metastatic disease. Studies suggest that screening tests performed in a comfortable and safe environment make for a positive experience and could potentially remedy the lack of screening (77–80). Positive feelings resulted from sensitive health care providers acknowledging patients’ vulnerability and considering the physical discomfort they may feel while simultaneously affirming their identities. Avoiding the use of gendered or binary language in screening, especially for many cancers is of note, for example, employing the use of “chest” or “upper body” instead of “breast” (48, 81). Simply naming the organ involved instead of referring to female/male reproductive cancer or other body parts is also consequential. Furthermore, health care providers may need to provide education about the need for cervical cancer screening for lesbian women, trans and non-binary people (48). Historically, there has been a misconception that lesbians did not need cervical cancer screening, and some health care providers and community members may still erroneously believe this to be true (48). Therefore, grassroots education for HCP is needed and many Canadian resources and guidelines are available online to this end, these promote preventive care, early diagnosis, and effective management (48). Specialized 2SLGBTQIA+ clinical groups focused specifically on the management of health conditions or the prevention and/or management of behavioral risk factors for chronic diseases have also been shown to be effective in improving care (82–90). A summary of evidence on the prevalence of health conditions can be found in Table 1.

**Table 1:** A summary of evidence on the prevalence of health conditions, outcomes, and behaviors among individuals from the LGBQ community and transgender individuals in comparison to the general adult population in Canada or the United-States of America (USA).

	LGQ or homosexual?	Bisexual	Transgender	General or heterosexual population	Reference
	% and/or OR	% or OR	% or OR	% or OR	
Health conditions					
Cardiovascular disease (USA) women men	5.0% 6.8%	7.0% 14.5%	ND	5.8% 7.5%	Blosnich et al. (239)
Cancer (USA) prostate cancer breast colon	3.4% 17.8% 3.6%	7.8% 13.3% 1.7%	ND	4.3% 20.6% 3.6%	Tamargo et al. (240)
Overweight or obesity (USA) women men	60.7% 52.6%	61% 57.3%	ND	54.3% 70%	Blosnich et al. (239)
Arthritis (USA) women men	50.3% **1.57** 28.9% **0.84**		ND	44.7% 34.2%	Fredriksen-Goldsen et al. (241)
Mood or anxiety disorder	17.4%	23.9%	ND	11.4%	Gilmour (242)
Depression	**2.09**	**3.73**	60%	**1**	Scott et al. (243) House of commons (4)
Diagnosed with a Sexually Transmitted Disease	**1.19**	**3.34**	ND	ND	Steele et al. (244)
Health outcomes					
Suicide ideation	5.4%	12.9%	36%	2.3%	Gilmour (242)
Perception of health (poor or fair)	**1.05**	**2.15**	ND	**1**	Steele et al. (244)
Health behaviors					
Substance use	**2.67**	**2.00**	ND	**1**	Steele et al. (244)
Smoking	**1.77**	**2.04**	ND	**1**	Steele et al. (244)

OR, odds ratio; ND, no data. Values in bold are Odds Ratio.

### 3.1. The transgender population

The transgender (trans) community is among the most neglected and underserved populations in the 2SLGBTQIA+ community. Marginalization, discrimination, lack of HCP experience and knowledge, inadequate services, and structural barriers are causal factors (91–96). Canadian studies have shown that more than half of trans participants have had negative experiences within the health care system, one-third have not been helped, and long wait times have been observed (97–101). In addition, trans women are more likely than trans men to go without medical treatment (102). Transgender individuals have seen their treatments delayed and have been subject to physical and verbal violence at a more alarming rate (32, 49, 103).

Trans people may decide to transition socially, transition medically, or not transition. Social transition refers to the cosmetic, social, and legal changes of transition, such as changing one’s appearance and name. A medical transition may involve hormone therapy and/or surgical procedures. Primary care needs are largely related to medically supervised transition and access to and control of hormone administration and dosing (104). Importantly, transition in many cases is essential, non-elective, and mitigates the risks of suicide and psychological distress prevalent in this population (99, 105–112). As an example, a cross-sectional study of a large Australian cohort found that 57% of transgender individuals were diagnosed with depression and 39% with anxiety (113). In addition to discrimination, the lack of trans-specific medical training has been identified as a major barrier to providing health care to this population (92–94, 114–118). In fact, only one-third of medical schools in Canada and the United States provide education on hormone therapy and surgical transition (119). In a national survey, only 10% of medical students felt they were prepared to care for trans patients (118). In Canada, primary care HCP serve the transgender population; however, the number of clinicians with the necessary skill set to support this population adequately remains low. As a result, patients are placed on waiting lists or are required to travel great distances, and incur costs related to travel and medical procedures to receive adequate health care (100). In addition, general practitioners often refer patients to specialists, such as endocrinologists, often by lack of experience and because they presume the patients will be in better hands. This approach can be interpreted as an unwillingness on the part of the HCP to provide care to the patient and can also increase wait time (93). Incidentally, the great majority (80%) of endocrinologists have not received trans-specific training (120). Another financial barrier is the consultation with a private HCP (psychologists, psychotherapists, or social workers). This step is mandatory for the patient to obtain a letter recommending gender affirmation surgery and is not always straightforward (4). Instead, the Canadian Professional Association for Transgender Health now recommends that informed consent be promoted for a more effective and timely service (121). Prompt access to treatment could greatly reduce the distress felt by this at-risk population (4).

Current curricula do not provide the knowledge required to care for trans people adequately. To fill the knowledge gap in Canada, organizations such as TransCare BC, Rainbow Health Ontario and the Canadian Professional Association for Transgender Health have put together conferences and workshops specifically tailored to providing primary care to trans people (122). Indeed, Canadian primary care guidelines from Ontario and British Columbia are available online for clinicians (98, 123–125). Another example of online continuing education is the Ontario Ministry of Health and Long-Term Care funded program, Trans Health Connection, which provides HCP with training on cultural competency and clinical care for the trans population (98). Finally, excellent guidelines are also freely available from the Registered Nurses’ Association of Ontario (48). Of note, much of the Canadian health care literature comes from the Ontario Trans PULSE study and may not be generalizable to the broader Canadian context due to provincial differences in health care services, such as health care coverage and access to surgeries (122). In Canada, the health system is publicly financed; most health expenditures are financed through the general tax revenues of the federal, provincial, and territorial governments. While the federal government has a role in setting national standards for funding, streamlining data collection and research as well as regulating prescription drugs, Canadian provinces and territories are responsible for financing as well as administrating their own tax-funded and universal hospital and medical care plans (126). This could explain inter-province variability in service delivery and accessibility for the 2SLGBTQIA+ community. Notably, Ontario has the highest number of health programs that target the 2SLGBTQIA+ community (29.5%). That proportion is higher than the programs available across the country, and especially in comparison to the Atlantic provinces. That means, in addition to the variations in logistics between provinces, there are also important variations in programs available to provide health care to individuals from the 2SLGBTQIA+ community. In 2019, an environmental scan of programs in Canada revealed these substantial variations as illustrated in Table 2 (57).

**Table 2:** Environmental scan of programs targeting sexual and gender minority populations in Canada (2019).

Geographical region	*n*	%
Alberta	29	13.2
British Columbia	27	12.3
Manitoba	15	6.8
New Brunswick	3	1.4
Newfoundland and Labrador	3	1.4
Northwest Territories	2	0.9
Nova Scotia	9	4.1
Nunavut	0	0
Ontario	65	29.5
Prince Edward Island	2	0.9
Quebec	45	20.5
Saskatchewan	3	1.4
Yukon	1	0.5
Canada	16	7.3
Total	220	100

*n* represent the number of specialized programs in each geographical region.

% represents the proportion of specialized programs by geographical region to illustrate the uneven distribution throughout Canada.

### 3.2. Two-Spirit peoples

We use the designation ‘Indigenous’ in the present document to describe individuals and groups who identify as descendants of civilizations that predate colonization in Canada. Although there is no universal definition that has been accepted to describe this extremely diverse group of peoples, we employ this terminology because it is understood that individuals and communities will be supported in self-defining what it means to them (127).

Colonization and racism are intertwined and have resulted in deep-rooted and continuing stigma against Indigenous peoples, which have a demonstrably negative impact on health and well-being (128, 129). Racist ideologies have pervaded education, housing, food securities and employment, societal systems and institutions including child welfare, the criminal justice system and, notably, health care (127). The most egregious barrier to health care for Indigenous peoples remains attitudinal and systemic racism (130). Indeed, research shows that racism is so prevalent that Indigenous peoples strategize around anticipated racism before visiting health care facilities or avoid them altogether (131–133). Past horrific assimilationist practices, namely residential schools, the Sixties Scoop, and contemporary child welfare, have also resulted in deep-rooted intergenerational trauma negatively affecting well-being and health (134, 135).

Two-Spirit is a contemporary umbrella term to describe Indigenous peoples with both masculine and feminine spirits outside of the Western colonialist framework (136, 137). Although a unifying term, many Indigenous tribes have their own words, definitions, and cultural understanding of what this means to them; western categories do not accurately reflect the ontologies of gender and sexuality for Indigenous peoples. Oral histories show that Two-Spirit peoples held important roles within Indigenous spirituality and were honored and respected. They were indeed vital to the collective well-being and survival of their tribes, contributing to the maintenance of Indigenous legal, cultural, and spiritual systems (135). Indigenous peoples belonging to the 2SLGBTQIA+ are particularly vulnerable to health inequities due to intersectional forms of discrimination and invisibility within the health care system (138, 139). Consequently, the experiences of Two-Spirit peoples are unique. There is however a scarcity of literature that look exclusively at the health and health inequities of Canadian Two-Spirit peoples (138). And so, the Canadian House of Common’s Standing Committee on Health has called for increased research and programing for the community. It is particularly important for HCP and health care institutions to work toward reconciliation and decolonization to ameliorate existing inequities (140) and acknowledge the Calls to Action and Calls for Justice. Notably, the resiliency of Indigenous peoples has allowed them to survive and flourish in the face of horrendous colonial oppression, demonstrating collective strength and fortitude. There is much to learn here for the betterment of health and health care, not only for Two-Spirit peoples but for the broader LGBTQIA+ community. Understanding the historical, contemporary, and emergent issues faced by Indigenous and Two-Spirit peoples must be a Canadian priority (135).

## 4. Education and training of health care professionals

HCP have reported a lack of curricula geared toward the 2SLGBTQIA+ community (119, 141–145), and HCP who have not been trained appropriately feel that their lack of training negatively affect their ability to care for patients (19, 146, 147). Notably, a survey conducted at the University of Ottawa found that 41% of medical students witnessed anti-2SLGBTQIA+ attitudes within the education system (148). Given the lack of ingrained 2SLGBTQIA+ cultural competency and sensitivity, when in an uncomfortable situation with a member of the community, HCP tend to disconnect from patients which could be perceived as heterosexism by consulting patients (10, 146). This real or perceived disconnect between patients and HCP negatively impacts the consultation and treatments (41, 42). However, when education and training are available, these positively influence comfort levels and diminish anxiety in students and health care providers (149–152). It would thus be imperative to normalise and require HCP to be trained in matters of cultural competency, and the specific health care needs of 2SLGBTQIA+ individuals (19, 141, 145–147, 153–155). Nevertheless, despite overt efforts to deliver equity of care and with no remaining explicit prejudices, discrimination is still common and can be explained by the resilience of unconscious, implicit biases (41, 42, 149, 156–170). Implicit biases are not as easily modifiable as explicit biases because they are firmly entrenched beliefs; thus, for significant change to occur, dedication on the part of the learner is required as well as a strong educational support. Because measures of implicit biases are more strongly associated with real-world behaviors than explicit biases (171, 172), the former should be the focus of educational practices. Furthermore, with less time and limited information processing capacity, providers’ decisions are increasingly governed by implicit stereotypes and biases (173, 174). Moving beyond cultural competency and including training on implicit biases is thus undeniably important (41, 42, 158, 159).

Educating HCP on implicit bias as well as culturally competent care such as 2SLGBTQIA+ social issues and health should be integrated into curricula, in continuing education and in all learning and teaching opportunities when health assessment, health promotion and disease prevention are addressed (43, 44, 175). These themes should not only be promoted but integrated into multimodal learning strategies, experiential learning (e.g., case studies, role-playing, simulation) as well as reflective practice to better prepare HCP to meet the health care needs of 2SLGBTQIA+ patients (60, 147, 176). Moreover, because HCP have generally not had proper training, health care authorities should include and require training for all new employees and offer opportunities for continued education (142, 165, 177). Interventions geared toward training programs have been highlighted, show good promise, and are briefly summarized in four points:

- A good first step could be to evaluate the inclusivity of programs/institutions using tools such as the Health care Equality Index (HEI) benchmark (178). Although certification is only available in the United States, they offer numerous learning resources and a scoring system methodology online that could be reproduced for use in Canada (179).
- Include implicit bias training in teaching programs, such as case scenarios used by the Gay and Lesbian medical Association and Fenway Institute (180). Practicing self-reflection and awareness through journaling, self-assessment, deconstructing existing beliefs and analyzing social issues affecting the lives of the 2SLGBTQIA+ community have been shown to be effective tools (181, 182). Programs incorporating bias training should also evaluate their efficacy with tests such as the Implicit Association Test (IAT) (183).
- A training program focused on cultural competency, the inclusion of 2SLGBTQIA+ perspectives and patient-centered care has been shown to be efficacious (46). The trainings found in the literature use a range of effective learning strategies to increase cultural competence, such as lectures, readings, videos, interviews, or presentations by people from the community (intergroup contact), and group discussions. Of these strategies, intergroup contact, i.e., the inclusion of 2SLGBTQIA+ perspectives in training institutions, is most effective in promoting more tolerant attitudes toward this population (46). In these training programs, a variety of topics were covered, including sexual orientation, gender identity, sexual history, 2SLGBTQIA+ terminology, disclosure of orientation and gender identity, discrimination and bias, the impact of discrimination on health, factors affecting patient access and medical care, myths and stereotypes, medical care for transgender people, and legal concerns. Even more importantly, repetition of relevant trainings was a strategy that increased the long-term comfort level of HCP (46).
- The inclusion of cultural safety in teaching programs has been proposed (47, 48). Cultural safety moves away from a focus on cultural differences (cultural competence) to a view of the health system environment as a site of change (184), that involves understanding the history, safety needs, power imbalances (oppression), and the influence of staff values and beliefs on service delivery (185, 186). Research has identified several key components in cultural safety interventions (47), these include provider self-reflection, addressing bias and discrimination, and patient-centered care such as building authentic relationships with patients, power sharing, validation of patient autonomy/intuition, and meaningful training for HCP (127, 184, 187, 188).
  There is presently no national standard for health care education in Canada (189). Although several focus points and potential solutions have been highlighted here, no Canadian guidelines or resources exist to transform the current lack of 2SLBGTQIA+ curricula. Data-backed interventions and evaluation of their efficacy are urgently needed for nation-wide implementation.

## 5. System and infrastructure

Cis normativity, the concept of two distinct and opposing genders (women and men), and heteronormativity are profoundly embedded in our society and are reflected in our physical environment. Western Euro-Christian cultural beliefs about gender are being challenged and we must address the inadequacies of our environment to meet the needs of all people, regardless of their gender and sexual orientation. To create a supportive environment, HCP must first assess their own belief systems, cultural norms, and biases to increase their cultural sensitivity, and develop better relationships with 2SLGBTQIA+ patients and gain their trust (Supplementary Table S1) (190).

Creating an open and safe space in all levels of the health care system is essential. In addition, waiting rooms, bathrooms, common areas, and patient care spaces should promote inclusion and support of 2SLGBTQIA+ patients and families through the display of a non-discrimination policy, pro-2SLGBTQIA+ symbols, magazines, posters, information, decorations, or images depicting 2SLGBTQIA+ families (77, 191–198). For instance, bathrooms are an integral and necessary part of our daily lives (199). Gender-neutral bathrooms should be available, and people should also be able to use bathrooms that conform to their gender identity (48, 200). It is therefore recommended to provide gender-neutral and gender-specific toilets and most importantly, to have educated staff who can defend the rights of their users in case of discrimination (Supplementary Table S1) (48). Of note, experts have warned against signage in unsafe spaces, for instance, where employees have not been properly trained to deal with 2SLGBTQIA+ patients or instances of discrimination (48). Visibility can only happen in safe spaces; hence, it is important to have adequate training, and anti-discrimination policies that address instances of discrimination and promote accountability (201, 202).

To open the lines of communication between patients and HCP, electronic medical forms and records should be inclusive and allow for disclosure of sexual orientation and gender and contain neutral language that allows the patient to openly self-identify without presumption (54, 93, 200). At a minimum, all medical forms, processes, language/terminology, and records should include the individual’s chosen name, pronouns, gender identity, sex assigned at birth and sexual orientation (Supplementary Table S1). Assuming a patient’s pronouns based on appearance is harmful because a stereotype regarding gender expression is implicitly reinforced. In fact, this disclosure of the information is of great importance and will have a positive impact on the patient’s health and will make the patient feel comfortable and safe, if done properly (10, 15, 36, 54, 192, 193, 195, 203–205). It is also good practice for HCP to declare their own pronouns before asking the patient to share theirs (206), all the while maintaining privacy when information is shared (194, 207–209). It is important to be non-judgmental and comfortable when asking questions about gender, sex, sexuality, and sexual activity (77, 194, 208).

Moreover, the broader political environment can influence the success of institutional initiatives for change (210), such as bathroom inclusivity and gender neutrality in medical forms and in communication. Organizational cultures influence the acceptability of discriminatory and stigmatizing practices and how HCP interact with patients (Supplementary Table S1). Injurious practices can become embedded in the culture of health care organizations and be reinforced by clinical and non-clinical staff. This can manifest itself in stigmatizing language, assumptions, lack of confidentiality, and denying care or access to treatment. Thus, structural change needs to be accompanied with proper training of staff, accountability, and well cemented inclusive organizational culture.

## 6. Discussion

The drivers of discrimination and stigma impacting 2SLGBTQIA+ health must be addressed at the organizational level on an evidentiary basis (47, 184, 211–215). Interventions should target multiple levels simultaneously, such as creating inclusive physical environments, enhancing workforce diversity, policy changes, practice changes, with education of both clinical and nonclinical staff being the bedrock of change (202, 212, 216–218). A better understanding of health disparities unique to each individual group within the community, notably the trans and bisexual community, and considering intersectionality will be crucial to better tailor service delivery. This will require national data collection and funding large scale research projects geared toward understanding the 2SLGBTQIA+ health care experience. The latter could include descriptive studies of patient journey as well as the development and validation of educational programs tackling implicit bias. Lack of data, lack of national educational programs and guidelines, and the scarce investment in change are presently hindering or impeding forward movement. The lack of national guidelines and best practices are reflected in inter-province variability in terms of programs offered for the health and well-being of sex and gender minorities, with a particularly deplorable amount offered in the Maritime provinces as compared to Ontario. Realistically, overall change will take some time. It is thus important to aim at firstly armoring the 2SLGBTQIA+ community with tools to navigate the health care system. Reducing the impact of stigma on well-being can be achieved through psychoeducation programs at the individual level (219–221); which could help members of the community better navigate and cope with an imperfect health care system. These interventions can also take place in a group setting and include cognitive and behavioral components, such as cognitive-behavioral therapy, and aim to change internalized discriminatory and stigmatizing beliefs, improve coping skills, promote autonomy, and enhance social support (219–225).

To address gender related health inequalities in the past, the World Health Organization and the United Nations stressed the importance of transforming all areas of the health sector to integrate the gender perspective (226–228). This integral change was to encompass actions on policy, research, including interventions at the individual level (training/education). However, more than 20 years of research reveals that gender inequities remain embedded in health systems, and unconscious gender bias and sexism still have a pernicious impact on patient care (229–231). Over the years, institutions have seldom invested in education/training, data collection, changes in workplace culture, and human resource management to make sex and gender equality goals and standards an integral part of their governance (232). Sex and gender inclusivity had become an idea that has engrossed many institutions and governments at the expense of substantial actions to remedy health inequities (232–234). Lack of awareness and capacity in policy making, underfunding, bureaucratization, lack of evidence, and lack of patient participation in decision making are noted as pitfalls (235). A close parallel can be drawn between the struggle for gender inclusion in medicine and the inclusion of the 2SLGBTQIA+ community. Indeed, this could present an opportunity to learn from the mistakes of the past, move forward and avoid making the same missteps in addressing the health inequality of the 2SLGBTQIA+ community. Investing in diligent and exhaustive research-backed interventions at the organizational level could avoid such a misuse of resources and precious time (233, 235).

It is also important to highlight that gender inequities do not only have an impact on the 2SLGBTQIA+ community but is also endemic to the cis-gender and heterosexual population. Restrictive gender norms maintain a hierarchical system in which dominant forms of masculinity are favored. A gender system is created that not only undermines the health and human rights of girls and women, but also promotes the marginalization and discrimination of all those who transgress restrictive gender norms, including boys and men (230, 236–238). Ultimately, both face the same misogynistic and gendered barriers, therefore, the inclusion of the 2SLGBTQIA+ community in health care stretches its reach beyond this to all who are subjugated.

## 7. Conclusion

2SLGBTQIA+ inequities in the Canadian Health care system need to be corrected. Systemic discrimination, patient care and access to care need to be addressed through large scale national education and training programs with research and data collection being the linchpin of all forward movement and progress, all of which will require significant investment.

## 8. Search methodology

A literature review was performed using PubMed, Embase, and CINAHL. A broad subset of keywords relating to Canadian health care for sexual and gender minorities were used and Canadian articles were prioritized. Canadian grey literature such as federal and provincial government reports, health care authorities and other community-based communications were included in our search.

## Author contributions

DC reviewed the literature and wrote the manuscript under the supervision of NB. CJ contributed to the writing and editing of the manuscript. NB wrote sections, edited, and corrected the manuscript. All authors reviewed, provided feedback, and approved the final version of the manuscript.

## Conflict of interest

The authors declare that the research was conducted in the absence of any commercial or financial relationships that could be construed as a potential conflict of interest.

## Publisher’s note

All claims expressed in this article are solely those of the authors and do not necessarily represent those of their affiliated organizations, or those of the publisher, the editors and the reviewers. Any product that may be evaluated in this article, or claim that may be made by its manufacturer, is not guaranteed or endorsed by the publisher.
