# Supplementary material for: Review of current 2SLGBTQIA+ inequities in the Canadian health care system
Source: Front Public Health. 2023 Jul 18;11:1183284. doi: 10.3389/fpubh.2023.1183284 (PMC10392841; doi:10.3389/fpubh.2023.1183284)
Supplement: Supplementary file 1 [file Table_1.DOCX]

**Supplementary Table 1:** Summary of systemic and infrastructural changes

|  |  |
| --- | --- |
| hcp tRAINING | Adequate HCP training (e.g., cultural competency, implicit bias training, cultural safety).  ****See section 4:*** *Education and training of health care professionals*. |
| safer spaces | 1. Promote inclusion and support of 2SLGBTQIA+ patients and families through the display of a non-discrimination policy, pro-2SLGBTQIA+ symbols, magazines, posters, information, decorations, and/or images depicting 2SLGBTQIA+ families. 2. Gender neutral and gender specific bathrooms should be available.   **Note:** *Visibility can only happen in* ***safe spaces****; hence, it is important to have adequate training for both clinical and non-clinical staff, and anti-discrimination organizational policies that address instances of discrimination and promote accountability.* |
| Inclusive communication | 1. Inclusive electronic medical forms and records that allow for disclosure of sexual orientation and gender should include neutral language that allows the patient to openly self-identify without presumption. 2. All medical forms, processes, language/terminology, and records should include the individual’s chosen name, pronouns, gender identity, sex assigned at birth and sexual orientation. |
| Organizational culture | Organizational leadership teams (i.e., upper-level executives) determine the acceptability of discriminatory and stigmatizing practices. Leadership teams should take an open and strong stance against instances of discrimination and actively promote institutional initiatives for change. |

*For more detailed recommendations for infrastructural changes, please refer to available Best Practices guidelines from the Registered Nurses’ Association of Ontario* [*https://rnao.ca/sites/rnao-ca/files/bpg/2SLGBTQI_BPG_FIINAL_WEB_2.0.pdf*](https://rnao.ca/sites/rnao-ca/files/bpg/2SLGBTQI_BPG_FIINAL_WEB_2.0.pdf) *(48)*
